# Supplementary material for: Efficacy of Statin Treatment According to Baseline Renal Function in Korean Patients with Acute Myocardial Infarction Not Requiring Dialysis Undergoing Newer-Generation Drug-Eluting Stent Implantation
Source: J Clin Med. 2021 Aug 9;10(16):3504. doi: 10.3390/jcm10163504 (PMC8396958; doi:10.3390/jcm10163504)
Supplement: Supplementary file 1 [file jcm-10-03504-s001.zip › jcm-1326910-supplementary.pdf]

Efficacy of statin treatment according to baseline renal function in Korean patients with acute myocardial infarction not requiring dialysis undergoing newer-generation drug-eluting stent implantation

Running title: Effects of statin in AMI

Yong Hoon Kim[1,*], Ae-Young Her[1], Myung Ho Jeong[2], Byeong-Keuk Kim[3], Sung-Jin Hong[3], Seunghwan Kim[4], Chul-Min Ahn[3], Jung-Sun Kim[3], Young-Guk Ko[3], Donghoon Choi[3], Myeong-Ki Hong[3] and Yangsoo Jang[3]

Supplementary Online Contents

Table S1. Univariate analysis for MACE.

Table S2. Hazard ratios for the 2-year major clinical outcomes in statin nonusers

Table S3. Independent predictors for MACE and all-cause death in statin nonusers.

Table S1. Univariate analysis for MACE.

Variables	Statin users		Statin nonusers		Total	
	HR (95% CI)	*p*	HR (95% CI)	*p*	HR (95% CI)	*p*
Male	1.325 (1.157 - 1.518)	<0.001	1.105 (0.872 - 1.400)	0.407	1.227 (1.135 - 1.436)	<0.001
Age	1.022 (1.017 - 1.027)	<0.001	1.026 (1.017 - 1.036)	<0.001	1.024 (1.019 - 1.028)	<0.001
LVEF	0.978 (0.973 - 0.984)	<0.001	0.961 (0.953 - 0.970)	<0.001	0.972 (0.967 - 0.977)	<0.001
BMI	0.960 (0.941 - 0.980)	<0.001	0.938 (0.906 - 0.972)	<0.001	0.951 (0.934 - 0.968)	<0.001
SBP	0.997 (0.994 - 0.999)	0.007	0.992 (0.989 - 0.996)	<0.001	0.995 (0.993 - 0.997)	<0.001
DBP	0.994 (0.990 - 0.998)	0.003	0.989 (0.983 - 0.996)	0.001	0.992 (0.989 - 0.995)	<0.001
Cardiogenic shock	1.431 (1.074 - 1.907)	<0.001	1.831 (1.259 - 2.664)	0.002	1.626 (1.295 - 2.041)	<0.001
CPR on admission	2.680 (2.132 - 3.368)	<0.001	5.461 (4.157 - 7.172)	<0.001	3.560 (2.994 - 4.232)	<0.001
Killip class III/IV	2.398 (2.044 - 2.813)	<0.001	3.227 (2.605 - 4.122)	<0.001	2.754 (2.419 - 3.136)	<0.001
STEMI	1.300 (1.148 - 1.473)	<0.001	1.087 (0.877 - 1.348)	0.445	1.226 (1.101 - 1.366)	<0.001
Primary PCI	1.646 (0.905 - 2.992)	0.102	1.330 (0.625 - 2.830)	0.459	1.461 (0.914 - 2.333)	0.113
PCI within 24 hours	1.021 (0.794 - 1.314)	0.870	1.231 (0.825 - 1.838)	0.308	1.106 (0.801 - 1.557)	0.582
Hypertension	1.498 (1.320 - 1.700)	<0.001	1.194 (0.964 - 1.479)	0.104	1.419 (1.273 - 1.582)	<0.001
Diabetes mellitus	1.791 (1.575 - 2.036)	<0.001	1.613 (1.298 - 2.004)	<0.001	1.780 (1.594 - 1.988)	<0.001
Dyslipidemia	1.089 (0.905 - 1.312)	0.366	1.088 (0.756 - 1.566)	0.650	1.053 (0.893 - 1.243)	0.538
Previous MI	1.755 (1.364 - 2.256)	<0.001	2.119 (1.375 - 3.265)	0.001	1.797 (1.446 - 2.333)	<0.001
Previous PCI	1.499 (1.202 - 1.870)	<0.001	1.488 (1.029 - 2.153)	0.035	1.504 (1.244 - 1.819)	<0.001
Previous CABG	2.190 (1.136 - 4.222)	0.019	1.572 (0.391 - 6.311)	0.524	2.019 (1.115 - 3.654)	0.020
Previous HF	1.250 (0.690 - 2.266)	0.461	3.298 (2.076 - 5.240)	<0.001	2.248 (1.565 - 3.228)	<0.001
Previous CVA	1.909 (1.558 - 2.339)	<0.001	1.986 (1.433 - 2.754)	<0.001	1.958 (1.648 - 2.327)	<0.001
Current smoker	1.309 (1.150 - 1.489)	<0.001	1.565 (1.243 - 1.969)	<0.001	1.378 (1.232 - 1.542)	<0.001
Peak CK-MB	1.000 (0.999 - 1.002)	0.729	1.001 (1.000 - 1.002)	0.454	1.000 (0.999 - 1.002)	0.923
Peak troponin-I	1.001 (1.000 - 1.003)	0.650	1.002 (1.001 - 1.003)	0.002	1.001 (1.001 - 1.003)	0.358
NT-ProBNP	1.002 (0.999 - 1.004)	<0.001	1.002 (1.000 - 1.003)	<0.001	1.002 (0.999 - 1.004)	<0.001
High-sensitivity CRP	0.999 (0.997 - 1.001)	0.379	1.000 (0.997 - 1.003)	0.987	1.000 (0.998 - 1.001)	0.578

Serum creatinine	1.055 (1.037 - 1.073)	<0.001	1.113 (1.068 - 1.159)	<0.001	1.061 (1.047 - 1.075)	<0.001
eGFR	0.990 (0.988 - 0.992)	<0.001	0.990 (0.987 - 0.994)	<0.001	0.989 (0.988 - 0.991)	<0.001
Blood glucose	1.002 (1.001 - 1.003)	<0.001	1.003 (1.002 - 1.004)	<0.001	1.003 (1.002 - 1.003)	<0.001
Total cholesterol	0.997 (0.995 - 0.998)	<0.001	0.991 (0.989 - 0.994)	<0.001	0.995 (0.994 - 0.996)	<0.001
Triglyceride	0.999 (0.998 - 1.000)	0.008	0.999 (0.997 - 1.000)	0.032	0.999 (0.998 - 1.000)	<0.001
HDL	0.990 (0.984 - 0.995)	<0.001	0.963 (0.953 - 0.974)	<0.001	0.983 (0.978 - 0.988)	<0.001
LDL	0.998 (0.996 - 0.999)	0.006	0.991 (0.988 - 0.995)	<0.001	0.996 (0.994 - 0.997)	<0.001
Discharge medications						
Aspirin	2.527 (1.491 - 4.283)	0.001	1.171 (0.686 - 2.000)	0.562	2.082 (1.432 - 3.028)	<0.001
Clopidogrel	1.127 (0.928 - 1.368)	0.229	1.802 (1.327 - 2.447)	<0.001	1.014 (0.861 - 1.195)	0.865
Ticagrelor	1.134 (0.874 - 1.471)	0.344	2.799 (1.605 - 4.880)	<0.001	1.136 (0.898 - 1.438)	0.287
Prasugrel	1.344 (0.972 - 1.859)	0.074	1.735 (0.820 - 3.668)	0.149	1.178 (0.916 - 1.514)	0.201
Cilostazole	1.033 (0.881 - 1.211)	0.691	1.451 (1.057 - 1.991)	0.021	1.105 (0.918 - 1.351)	0.439
ACEI	1.542 (1.360 - 1.747)	<0.001	1.491 (1.186 - 1.874)	0.001	1.616 (1.450 - 1.802)	<0.001
ARB	1.427 (1.252 - 1.626)	<0.001	2.153 (1.573 - 2.946)	<0.001	1.124 (0.999 - 1.265)	0.051
BB	1.360 (1.153 - 1.560)	<0.001	2.301 (1.858 - 2.850)	<0.001	1.677 (1.480 - 1.901)	<0.001
CCB	1.223 (0.961 - 1.557)	0.102	1.139 (0.717 - 1.810)	0.582	1.151 (0.919 - 1.411)	0.234
Statin, n (%)						
Atorvastatin	1.066 (0.941 - 1.207)	0.319				
Rosuvastatin	1.129 (0.982 - 1.299)	0.088				
Simvastatin	1.437 (1.047 - 1.972)	0.025				
Pitavastatin	1.340 (1.094 - 1.640)	0.005				
Pravastatin	1.291 (0.810 - 2.058)	0.283				
Fluvastatin	2.108 (0.945 - 4.704)	0.069				
Infarct-related artery						
Left main	1.922 (1.358 - 2.719)	<0.001	3.048 (1.896 - 4.901)	<0.001	2.216 (1.675 - 2.931)	<0.001
LAD	1.040 (0.918 - 1.179)	0.534	1.279 (1.033 - 1.584)	0.024	1.107 (0.972 - 1.214)	0.428
LCx	1.120 (0.942 - 1.331)	0.200	1.263 (0.923 - 1.729)	0.145	1.158 (0.996 - 1.348)	0.057
RCA	1.043 (0.914 - 1.189)	0.535	1.344 (1.063 - 1.701)	0.014	1.143 (0.985 - 1.352)	0.304
Treated vessel						

Left main	1.775 (1.332 - 2.365)	<0.001	3.132 (2.068 - 4.744)	<0.001	2.037 (1.609 - 2.579)	<0.001
LAD	1.120 (0.986 - 1.273)	0.081	1.422 (1.140 - 1.774)	0.002	1.174 (1.051 - 1.311)	0.005
LCx	1.100 (0.958 - 1.264)	0.177	1.008 (0.785 - 1.296)	0.948	1.060 (0.938 - 1.196)	0.351
RCA	1.132 (0.998 - 1.284)	0.054	1.255 (1.003 - 1.569)	0.047	1.040 (0.932 - 1.161)	0.480
ACC/AHA lesion type						
Type B1	1.194 (1.001 - 1.301)	0.219	1.341 (0.991 - 1.815)	0.058	1.304 (1.103 - 1.541)	0.072
Type B2,	1.270 (1.118 - 1.442)	<0.001	1.177 (0.939 - 1.476)	0.157	1.217 (1.090 - 1.359)	0.001
Type C	1.105 (0.974 - 1.254)	0.121	1.073 (0.864 - 1.332)	0.524	1.080 (0.968 - 1.204)	0.169
Extent of CAD						
Single-vessel	1.912 (1.677 - 2.180)	<0.001	1.340 (1.079 - 1.665)	0.008	1.758 (1.571 - 1.966)	<0.001
2-vessel	1.112 (0.973 - 1.205)	0.073	1.049 (0.827 - 1.330)	0.695	1.118 (0.996 - 1.256)	0.058
≥ 3-vessel	1.893 (1.656 - 2.164)	<0.001	1.534 (1.216 - 1.935)	<0.001	1.819 (1.620 - 2.043)	<0.001
Pre-PCI TIMI 0/1	1.071 (0.946 - 1.217)	0.275	1.058 (0.850 - 1.316)	0.612	1.052 (0.943 - 1.173)	0.366
Types of stent						
ZES	1.030 (0.905 - 1.172)	0.659	1.061 (0.854 - 1.319)	0.593	1.035 (0.926 - 1.157)	0.543
EES	1.131 (0.997 - 1.282)	0.055	1.001 (0.807 - 1.240)	0.995	1.104 (0.993 - 1.241)	0.107
BES	1.028 (0.863 - 1.225)	0.755	1.023 (0.730 - 1.434)	0.893	1.006 (0.862 - 1.175)	0.939
Others	1.063 (0.932 - 1.541)	0.481	1.300 (0.746 - 2.262)	0.354	1.034 (0.747 - 1.499)	0.712
IVUS	1.004 (0.857 - 1.177)	0.959	1.100 (0.826 - 1.466)	0.513	1.038 (0.903 - 1.192)	0.602
OCT	1.485 (0.797 - 2.769)	0.213	1.019 (0.254 - 4.092)	0.979	1.350 (0.765 - 2.383)	0.301
FFR	1.399 (0.726 - 2.698)	0.316	2.010 (0.647 - 6.250)	0.228	1.232 (0.698 - 2.175)	0.472
Stent diameter	0.667 (0.572 - 0.777)	<0.001	0.601 (0.464 - 0.779)	<0.001	0.651 (0.570 - 0.743)	<0.001
Stent length	1.009 (1.004 - 1.014)	<0.001	1.029 (1.021 - 1.036)	<0.001	1.021 (1.008 - 1.016)	<0.001
Number of stent	1.169 (1.090 - 1.255)	<0.001	1.236 (1.099 - 1.390)	<0.001	1.177 (1.107 - 1.250)	<0.001

LVEF, left ventricular ejection fraction; BMI, body mass index; SBP, systolic blood pressure; DBP, diastolic blood pressure; CPR, cardiopulmonary resuscitation; STEMI, ST-segment elevation myocardial infarction; PCI, percutaneous coronary intervention; NSTEMI, non-STEMI; MI, myocardial infarction; CABG, coronary artery bypass graft; HF, heart failure; CVA, cerebrovascular accidents; CK-MB, creatine kinase myocardial band; NT-ProBNP, N-terminal pro-brain natriuretic peptide; CRP, c-reactive protein; HDL, high-density lipoprotein; eGFR, estimated glomerular filtration rate; LDL, low-density lipoprotein; ACEIs, angiotensin converting enzyme inhibitors; ARBs, angiotensin receptor blockers; BBs, beta-blockers; CCBs, calcium channel blockers; LAD, left anterior descending coronary artery; LCx, left circumflex

coronary artery; RCA, right coronary artery; ACC/AHA, American College of Cardiology/American Heart Association; CAD, coronary artery disease; TIMI, thrombolysis in myocardial infarction; ZES, zotarolimus-eluting stent; EES, everolimus-eluting stent; BES, biolimus-eluting stent; IVUS, intravascular ultrasound; OCT, optical coherence tomography; FFR, fractional flow reserve.

Table S2. Hazard ratios for the 2-year major clinical outcomes in statin nonusers

	Hazard Ratio (95% CI) Unadjusted	*p*-value	Event rates at 2 years[a]	Hazard Ratio (95% CI) Adjusted[b]	*p*-value
**MACE**					
Group B1 vs.	-		9.0 %	-	-
Group B2	1.066 (0.800 – 1.419)	0.663	9.7 %	1.142 (0.809 – 1.612)	0.450
Group B3	1.979 (1.461 – 2.680)	<0.001	17.0 %	1.251 (0.821 – 1.907)	0.298
Group B4	4.200 (2.829 – 5.712)	<0.001	32.2 %	2.648 (1.526 – 4.596)	0.001
Group B2 vs. Group B3	1.857 (1.411 – 2.444)	<0.001		1.238 (0.872 – 1.758)	0.333
Group B2 vs. Group B4	3.823 (2.757 – 5.302)	<0.001		2.055 (1.297 – 3.254)	0.002
Group B3 vs. Group B4	2.021 (1.436 – 2.844)	<0.001		1.676 (1.056 – 2.661)	0.029
**All-cause death**					
Group B1 vs.			3.4 %		-
Group B2	1.510 (0.978 – 2.333)	0.063	5.2 %	1.347 (0.754 – 2.409)	0.315
Group B3	3.786 (2.459 – 5.830)	<0.001	12.4 %	2.014 (1.076 – 3.769)	0.029
Group B4	8.788 (5.523 – 13.98)	<0.001	27.3 %	6.891 (3.114 – 12.25)	<0.001
Group B2 vs. Group B3	2.514 (1.780 – 3.550)	<0.001		1.180 (0.739 – 1.885)	0.488
Group B2 vs. Group B4	5.878 (3.998 – 8.644)	<0.001		2.914 (1.681 – 5.050)	<0.001
Group B3 vs. Group B4	2.304 (1.573 – 3.374)	<0.001		2.091 (1.238 – 3.233)	0.006
**Cardiac death**					
Group B1 vs.			2.3 %		-
Group B2	1.727 (1.025 – 2.910)	0.040	4.0 %	1.607 (1.790 – 3.268)	0.191
Group B3	4.290 (2.554 – 7.206)	<0.001	9.5 %	2.201 (1.054 – 4.596)	0.036
Group B4	8.871 (5.035 – 15.63)	<0.001	19.0 %	8.727 (3.295 – 14.11)	<0.001
Group B2 vs. Group B3	2.484 (1.671 – 3.691)	<0.001		1.051 (0.607 – 1.819)	0.858
Group B2 vs. Group B4	5.137 (3.253 – 8.110)	<0.001		2.681 (1.400 – 5.135)	0.003
Group B3 vs. Group B4	2.050 (1.303 – 3.224)	0.002		2.022 (1.166 – 3.184)	0.014
**Recurrent MI**					
Group B1 vs.			2.2 %		-
Group B2	1.400 (0.741 – 2.643)	0.300	1.6 %	1.355 (0.662 – 2.773)	0.406
Group B3	1.358 (0.690 – 2.673)	0.375	3.1 %	1.069 (0.417 – 2.739)	0.890
Group B4	1.846 (0.737 – 4.625)	0.191	4.4 %	2.741 (0.802 – 9.370)	0.108
Group B2 vs. Group B3	1.912 (0.972 – 3.764)	0.061		1.188 (0.497 – 2.841)	0.698
Group B2 vs. Group B4	2.697 (1.076 – 6.760)	0.034		2.435 (0.779 – 6.316)	0.126
Group B3 vs. Group B4	1.408 (0.546 – 3.630)	0.479		1.853 (0.547 – 6.278)	0.322

**Any repeat revascularization**					
Group B1 vs.			4.0 %		
Group B2	1.059 (0.681 – 1.647)	0.798	4.2 %	1.155 (0.687 – 1.941)	0.587
Group B3	1.537 (0.933 – 2.531)	0.091	6.1 %	1.319 (0.652 – 2.388)	0.441
Group B4	1.971 (0.971 – 4.000)	0.060	7.9 %	1.183 (0.424 – 3.299)	0.748
Group B2 vs. Group B3	1.451 (0.916 – 2.296)	0.112		1.288 (0.723 – 2.196)	0.391
Group B2 vs. Group B4	1.856 (0.940 – 3.665)	0.075		1.032 (0.410 – 2.600)	0.947
Group B3 vs. Group B4	1.299 (0.633 – 2.666)	0.476		1.812 (0.597 – 5.506)	0.294

[a]Event rates at 2 years were calculated by Kaplan-Meyer analysis. [b]Adjusted model included male, age, LVEF, BMI, cardiogenic shock, CPR on admission, Killip class III/IV, STEMI hypertension, diabetes mellitus, previous MI, PCI, and CVA, current smoker, NT-ProBNP, blood glucose, total cholesterol, HDL-cholesterol, ACEI, ARB, BB, LM (IRA and treated vessel), ACC/AHA type B2 lesion, single-vessel disease, ≥ 3-vessel disease, stent diameter, stent length, and number of stent. Group A1, statin users and eGFR ≥ 90 mL/min/1.73m^2; Group A2, statin users and eGFR 60-89 mL/min/1.73m^2; Group A3, statin users and eGFR 30-59 mL/min/1.73m^2; Group A4, statin users and eGFR < 30mL/min/1.73m^2; Group B1, statin nonusers and eGFR ≥ 90 mL/min/1.73m^2; Group B2, statin nonusers and eGFR 60-89 mL/min/1.73m^2; Group B3, statin nonusers and eGFR 30-59 mL/min/1.73m^2; Group B4, statin nonusers and eGFR < 30mL/min/1.73m^2; eGFR, estimated glomerular filtration rate; CI, confidence interval; LVEF, left ventricular ejection fraction; BMI, body mass index; CPR, cardiopulmonary resuscitation; STEMI, ST-segment elevation myocardial infarction; MI, myocardial infarction; PCI, percutaneous coronary intervention; CVA, cerebrovascular accident; HDL, high-density lipoprotein; ACEI, angiotensin converting enzyme inhibitor; ARB, angiotensin receptor blocker; BB, beta blocker; ACC/AHA, American College of Cardiology/American Heart Association

Table S3. Independent predictors for MACE and all-cause death in statin nonusers.

	MACE				All-cause death			
	**Unadjusted**		**Adjusted**		**Unadjusted**		**Adjusted**	
**Variables**	**HR (95% CI)**	***P* value**	**HR (95% CI)**	***P* value**	**HR (95% CI)**	***P* value**	**HR (95% CI)**	***P* value**
Group B1 vs. Group B2	1.066 (0.800 – 1.419)	0.663	1.109 (0.793 – 1.551)	0.545	1.510 (0.978 – 2.333)	0.063	1.313 (0.711 – 2.194)	0.125
Group B1 vs. Group B3	1.979 (1.461 – 2.680)	<0.001	1.216 (0.799 – 1.849)	0.361	3.786 (2.459 – 5.830)	<0.001	2.157 (1.785 – 3.312)	0.047
Group B1 vs. Group B4	4.200 (2.829 – 5.712)	<0.001	2.722 (1.447 – 5.120)	0.002	8.788 (5.523 – 13.98)	<0.001	5.596 (2.363 – 13.25)	<0.001
Group B2 vs. Group B3	1.857 (1.411 – 2.444)	<0.001	1.132 (0.807 – 1.586)	0.473	2.514 (1.780 – 3.550)	<0.001	1.750 (1.352 – 2.345)	0.474
Group B2 vs. Group B4	3.823 (2.757 – 5.302)	<0.001	1.781 (1.052 – 3.013)	0.032	5.878 (3.998 – 8.644)	<0.001	2.446 (1.303 – 4.591)	0.005
Group B3 vs. Group B4	2.021 (1.436 – 2.844)	<0.001	1.694 (0.999 – 2.690)	0.048	2.304 (1.573 – 3.374)	<0.001	1.945 (1.223 – 3.148)	0.030
Male	1.105 (0.872 – 1.400)	0.407	1.136 (0.846 – 1.526)	0.395	1.250 (0.929 – 1.683)	0.141	1.161 (0.799 – 1.688)	0.434
Age, ≥65 years	1.672 (1.342 – 2.083)	<0.001	1.283 (0.966 – 1.704)	0.085	2.292 (2.183 – 4.100)	<0.001	2.234 (1.474 – 3.385)	<0.001
STEMI	1.087 (0.877 – 1.348)	0.445	1.103 (0.857 – 1.419)	0.447	1.050 (0.796 – 1.386)	0.730	1.250 (0.892 – 1.752)	0.195
LVEF, <40%	2.861 (2.272 – 3.603)	<0.001	1.606 (1.200 – 2.149)	0.001	4.591 (3.475 – 6.065)	<0.001	2.415 (1.701 – 3.429)	<0.001
Cardiogenic shock	1.831 (1.259 – 2.664)	0.002	1.749 (1.039 – 2.435)	0.023	2.638 (1.735 – 4.009)	<0.001	2.489 (1.542 – 3.207)	0.006
CPR on admission	5.461 (4.157 – 7.172)	<0.001	3.822 (2.740 – 5.332)	<0.001	7.865 (5.747 – 10.76)	<0.001	3.691 (2.466 – 5.526)	<0.001
Hypertension	1.194 (0.964 – 1.479)	0.104	1.133 (0.878 – 1.462)	0.338	1.474 (1.114 – 1.948)	0.007	1.064 (0.757 – 1.494)	0.721
Diabetes mellitus	1.613 (1.298 – 2.004)	<0.001	1.157 (0.887 – 1.509)	0.282	1.764 (1.337 – 2.329)	<0.001	1.129 (0.791 – 1.611)	0.503
Previous heart failure	3.298 (2.076 – 5.240)	<0.001	1.350 (0.737 – 2.473)	0.331	3.533 (2.014 – 6.198)	<0.001	1.026 (0.486 – 2.164)	0.947
Current smokers	1.565 (1.243 – 1.969)	<0.001	1.287 (0.961 – 1.723)	0.091	1.971 (1.444 – 2.691)	<0.001	1.226 (0.828 – 1.816)	0.308
NT-ProBNP	1.002 (1.000 – 1.003)	<0.001	1.003 (1.001 – 1.004)	<0.001	1.001 (1.000 – 1.002)	<0.001	1.002 (1.001 – 1.003)	<0.001
Total cholesterol	0.991 (0.989 – 0.994)	<0.001	0.999 (0.992 – 1.006)	0.801	0.999 (0.986 – 0.992)	<0.001	1.004 (0.995 – 1.014)	0.368
Triglyceride	0.999 (0.997 – 1.000)	0.032	1.000 (0.998 – 1.001)	0.708	0.996 (0.994 – 0.998)	0.001	0.997 (0.995 – 1.000)	0.070
HDL-cholesterol	0.963 (0.953 – 0.974)	<0.001	0.971 (0.960 – 0.989)	0.364	0.945 (0.932 – 0.959)	<0.001	0.965 (0.949 – 0.982)	0.269
LDL-cholesterol	0.991 (0.988 – 0.995)	<0.001	0.994 (0.990 – 0.997)	0.001	0.991 (0.986 – 0.995)	<0.001	0.993 (0.983 – 1.003)	<0.001
Aspirin	1.171 (0.686 – 2.000)	0.562	1.074 (0.573 – 2.013)	0.825	1.713 (0.956 – 3.069)	0.071	1.103 (0.537 – 2.266)	0.790
Ticagrelor	2.799 (1.605 – 4.880)	<0.001	1.631 (0.839 – 3.168)	0.149	1.238 (0.460 – 3.335)	0.672	2.319 (0.557 – 9.658)	0.248
Prasugrel	1.735 (0.820 – 3.668)	0.149	1.480 (0.634 – 3.456)	0.364	1.504 (0.559 – 4.046)	0.419	1.644 (0.498 – 5.421)	0.414
ACEI	1.491 (1.186 – 1.874)	0.001	1.011 (0.760 – 1.346)	0.938	3.259 (2.274 – 4.672)	<0.001	1.763 (1.107 – 2.807)	0.017
BB	2.301 (1.858 – 2.850)	<0.001	1.951 (1.491 – 2.552)	<0.001	5.010 (3.700 – 6.783)	<0.001	3.276 (2.210 – 4.856)	<0.001
≥3-vessel disease	1.534 (1.216 – 1.935)	<0.001	1.347 (1.030 – 1.761)	0.030	1.736 (1.295 – 2.325)	<0.001	1.396 (0.987 – 1.975)	0.059
Stent diameter < 3.0mm	1.438 (1.146 – 1.804)	0.002	1.239 (0.952 – 1.613)	0.111	1.727 (1.300 – 2.294)	<0.001	1.434 (1.021 – 2.015)	0.038
Stent length ≥30mm	1.501 (1.189 – 1.895)	0.001	1.165 (0.888 – 1.527)	0.270	1.378 (1.016 – 1.868)	0.039	1.051 (0.733 – 1.509)	0.785

MACE, major adverse cardiac events; HR, hazard ratio; CI, confidence interval; Group B1, statin nonusers and eGFR ≥ 90 mL/min/1.73m^2; Group B2, statin nonusers and eGFR 60-89 mL/min/1.73m^2; Group B3, statin nonusers and eGFR 30-59 mL/min/1.73m^2; Group B4, statin nonusers and eGFR < 30mL/min/1.73m^2, eGFR, estimated glomerular filtration rate; STEMI,

ST-elevation myocardial infarction; LVEF, left ventricular ejection fraction, CPR, cardiopulmonary resuscitation; NT-ProBNP, N-terminal pro-brain natriuretic peptide; HDL, high-density lipoprotein; LDL, low-density lipoprotein; ACEI, angiotensin converting enzyme inhibitor; BB, beta-blocker.
